# High Blood Pressure-Associated Symptoms: Insights From a Population-Based Study in Pakistan

**DOI:** 10.7759/cureus.65446

**Published:** 2024-07-26

**Authors:** Salma Kidwai, Muhammad Haris, Abubaker Bilal, Sana Saleem, Haram Imran, Adnan Anwar, Atif A Hashmi

**Affiliations:** 1 Internal Medicine, Jinnah Sindh Medical University, Karachi, PAK; 2 Internal Medicine, Hamdard University Hospital, Karachi, PAK; 3 Internal Medicine, Jinnah Postgraduate Medical Centre, Karachi, PAK; 4 Internal Medicine, Jinnah Medical & Dental College, Karachi, PAK; 5 Physiology, Hamdard College of Medicine & Dentistry, Karachi, PAK; 6 Internal Medicine, Anabiya General Hospital, Karachi, PAK; 7 Pathology, Liaquat National Hospital and Medical College, Karachi, PAK

**Keywords:** hypertensive, normotensive, vision problem, headache, symptoms, hypertension, non-communicable disease

## Abstract

Background

All over the world, millions die of hypertension (HTN) every year. Given the influence of healthcare expenses, HTN represents a serious public health issue in developed and developing countries. HTN is common in Pakistan; however, there are several myths about the symptoms of raised blood pressure that need to be identified.

Objective

The objective of this study is to compare the frequency of high blood pressure-associated symptoms in normotensive and hypertensive adult populations in Karachi, Pakistan.

Methodology

A community-based cross-sectional observational study was conducted among 277 patients aged 18 years and above who were attending the OPD of different community health centers in Karachi, with and without HTN. Ethical approval was obtained, and data were collected using a convenient sampling technique on a predesigned questionnaire and analyzed using IBM SPSS Statistics for Windows, Version 20.0 (Released 2011; IBM Corp., Armonk, NY, USA).

Results

Out of the total study population, 88 (31.65%) were normotensive, and 189 (67.98%) were hypertensive. In the hypertensive group, approximately 100 (52.9%) were men and 89 (47.1%) were women. The mean ages of normotensives and hypertensives were 44.13 + 11.0 and 49.2 + 13.1 years, respectively. Mean age, heart rate, and smoking status were significantly different between the two groups. Among several perceived blood pressure symptoms like vision problems, sleep apnea, and abnormal heartbeat was significantly higher in the hypertensive group. Conversely, although headache, vertigo, edema, and epistaxis were more frequent in the hypertensive group, the difference was not statistically significant.

Conclusions

In our study, >65% of patients visiting OPD had high blood pressure. Several symptoms were found to be more prevalent in hypertensive individuals compared with non-hypertensive ones. More large-scale studies are recommended to further explore the common symptoms associated with HTN in our population.

## Introduction

Hypertension (HTN) is a major public health concern in the 21st century. HTN is referred to as systolic blood pressure (SBP) levels of 130 mm Hg and/or diastolic blood pressure (DBP) values rising to more than 80 mm Hg [1]. Despite being prevalent, observable, and curable, the illness is frequently asymptomatic and can lead to fatal consequences if left untreated. Over the last two decades, global population growth and aging have doubled the number of people with HTN. Over a billion people worldwide suffered from HTN in 2019, accounting for 82% of all hypertensive people living in low- and middle-income regions [2]. An approximate total of 8.5 million fatalities were attributed to HTN in 2015, encompassing complications such as ischemic heart disease, stroke, vascular issues, and renal dysfunction [3].

In a nationwide diabetes survey conducted in Pakistan between 2016 and 2017, HTN was reported in 46.2% of participants, of whom 44.3% were urban and 46.8% were rural. Punjab had the highest prevalence of hypertension at 49.2%, followed by Sindh at 46.3%, Balochistan at 40.9%, and Khyber Pakhtunkhwa at 33.3% [4]. Studies previously conducted in Pakistan showed a high prevalence of HTN based on lifestyle risk factors such as lack of physical activity, obesity, junk food consumption, unhealthy diet and sleeping habits, poor socioeconomic status, and lack of healthcare facility availability [5].

HTN is a threat that is not readily apparent because, in most cases, it does not manifest symptoms until it has progressed to a stage that is either severe or life-threatening. Because symptoms of HTN are often absent, the condition is frequently underdiagnosed, and therapeutic compliance is extremely low. Hypertensives are generally unaware of any clinical problems until they develop a heart attack, stroke, or kidney disease. They are diagnosed during a screening program or after an assessment of a seemingly unrelated condition [6].

Due to the limited availability of accurate symptoms of high blood pressure, assessing the connection between perceived symptoms and high blood pressure is challenging. The primary objectives of this research were to determine the frequency of symptoms that are associated with HTN.

## Materials and methods

Patient selection

This community-based, cross-sectional observational study was conducted at Anabiya General Hospital in Karachi, Pakistan. Patients included those who were aged 18 and above and attended the OPD of two different community health centers in Karachi for the duration of six months, from January to June 2023. Informed written consent was obtained from all the patients. Patient confidentiality was maintained by removing all patient-identifying information from the proformas. The study was approved by the Institutional Review Board of Anabiya General Hospital (Anabiya/22/081).

Patients visiting these centers for minor medical problems or high blood pressure were included in the study using a convenient sampling technique. These patients included both hypertensive and normotensive individuals with minor medical problems. A total of 277 patients were included in the study after excluding patients with diabetes, known cardiac issues, migraine, retinopathy, morbid obesity, and other severe illnesses. Similarly, all patients with severe medical or surgical problems were excluded. Information was recorded on a predesigned structured questionnaire about patients’ baseline characteristics, such as age, gender, height, weight, respiratory rate, heart rate, resting SBP and DBP, smoking status, and frequently perceived symptoms of HTN, including headache, vertigo, edema, chest pain, vision problems, dyspnea, epistaxis, increased urine output, nausea, sleep apnea, abnormal heartbeat or palpitation, fatigue, and confusion. The primary investigator/medical officer filled in all this information.

Patients were seated comfortably for three to four minutes before their blood pressure was measured using a mercury sphygmomanometer and stethoscope. Blood pressure was taken from both arms, and an average reading was entered.

Data analysis

All data were entered and analyzed using IBM SPSS Statistics for Windows, Version 20.0 (Released 2011; IBM Corp., Armonk, NY, USA). Calculations were performed to determine the means and standard deviations for continuous variables, as well as the frequencies and percentages for categorical variables. A p-value below 0.05 was deemed to indicate a significant difference between the two groups.

## Results

Baseline characteristics of normotensives and hypertensives

Table 1 shows the baseline characteristics of normotensive (n = 88, 31.65%) and hypertensive (n = 189, 67.98%) subjects. Altogether, there were 145 (52.3%) males and 132 (47.7%) females in this study. The overall gender distribution was similar across groups (normotensive: 45 (51%) male, hypertensive: 100 (52.9%) male); however, a slightly higher percentage of females were observed in the normotensive group (43; 48.9%) than the hypertensive group (89; 47.1%). The mean age of normotensives was 44.13 + 11.0 years, and that of hypertensives was 49.2 + 13.1 years, which is a statistically significant difference.

**Table 1:** Baseline characteristics in the normotensive and hypertensive adult populations of Pakistan All the values are presented as mean and standard deviation/frequency (percentage). The p-value represents the level of significance. * represents a statistically significant difference.

Variables		Normotensive (88)	Hypertensive (189)	p-value
Gender	Males	45 (51.0%)	100 (52.9%)	-
	Females	43 (48.9%)	89 (47.1%)	-
Age (years)		44.13 + 11.0	49.2 + 13.1	<0.01*
BMI (kg/m^2^)		26.87 + 4.9	26.85 + 6.2	>0.05
Respiratory rate(n/min)		23 + 6.5	23 + 3.4	>0.05
Heart rate (beats/min)		95 + 7.2	92 + 7.9	<0.01*
Smoker		3 (3.4%)	32 (16.9%)	<0.01*

BMI was similar between the groups (normotensive: 26.87 kg/m^2^, hypertensive: 26.85 kg/m^2^; p > 0.05). The respiratory rate was also similar in both groups (normotensive: 23 breaths/min, hypertensive: 23 breaths/min; p > 0.05). The group of individuals with high blood pressure had a heart rate that was considerably lower than the group of individuals with normal blood pressure (92 beats per minute vs. 95 beats per minute; p < 0.01). A significantly higher proportion of hypertensives were smokers compared to the normotensives (16.9% vs. 3.4%; p < 0.01).

Frequency of self-reported symptoms in normotensive and hypertensive individuals

Table 2 shows the frequency and association of symptoms with high blood pressure in the adult population of Pakistan. The study included both normotensive (n = 86) and hypertensive (n = 189) individuals. Hypertensive participants reported a higher percentage of headaches, with 151 individuals (79.9%) affected, compared to normotensive participants, who reported 63 cases (71.6%). Nevertheless, the difference did not approach statistical significance (p > 0.05). The prevalence of vertigo was 42 (47.7%) in individuals with normal blood pressure and 117 (61.9%) in individuals with high blood pressure. However, this difference was not found to be statistically significant (p > 0.05). Edema was present in approximately 36 (40.9%) of normotensive individuals and 81 (42.9%) of hypertensive individuals, which is not a significant difference (p > 0.05). Chest pain was reported by 35 (39.8%) of normotensive individuals and 94 (49.7%) of hypertensive individuals. Fatigue was prevalent in 61 (69.3%) of normotensive individuals and 138 (71.8%) of hypertensive individuals. Additionally, dyspnea was experienced by 43 (48.9%) of normotensive individuals and 104 (55.0%) of hypertensive individuals. However, upon statistical analysis, no significant difference was found (p > 0.05) for any of these symptoms.

**Table 2:** Symptoms of high blood pressure in normotensive and hypertensive groups All the values are presented as n (%). The p-value represents the level of significance. * represents a statistically significant difference.

	Study population		
Symptoms	Normotensive, n = 86	Hypertensive, n = 189	p-value
Headache	63 (71.6%)	151 (79.9%)	>0.05
Vertigo	42 (47.7%)	117 (61.9%)	>0.05
Edema	36 (40.9%)	81 (42.9%)	>0.05
Chest pain	35 (39.8%)	94 (49.7%)	>0.05
Fatigue	61 (69.3%)	138 (71.8%)	>0.05
Dyspnea	43 (48.9%)	104 (55.0%)	>0.05
Epistaxis	1 (1.1%)	8 (4.2%)	>0.05
Confusion	49 (55.7%)	126 (66.7%)	>0.05
Nausea	18 (20.5%)	50 (26.5%)	>0.05
Increased urine frequency	32 (36.4%)	86 (45.5%)	>0.05
Palpitation	20 (22.7%)	91 (40.1%)	<0.001*
Vision problem	35 (39.8%)	111 (58.7%)	<0.05*
Sleep apnea	15 (17.5%)	77 (40.7%)	<0.001*

Only one (1.1%) normotensive individual and eight (4.2%) hypertensive individuals reported epistaxis (nosebleeds), with no significant association (p > 0.05). Confusion was noted by 49 (55.7%) of those with normal blood pressure and 126 (66.7%) of individuals with high blood pressure. Nevertheless, there was no statistically significant difference (p > 0.05). Nausea was noticed by 18 (20.5%) of normotensive people and 50 (26.5%) of hypertensives, with no significant difference (p > 0.05).

The frequency of urination was higher in those with HTN (86; 45.5%) compared to those without HTN (32; 36.4%). However, this difference was not statistically significant (p > 0.05). Remarkably, sleep apnea was seen in 77 (40.7%) of HTN patients and 15 (17.5%) of normotensive patients. A difference of statistical significance was observed (p < 0.001). Palpitation or an abnormal heartbeat was observed in 20 (22.7%) of normotensive individuals and 91 (40.1%) of hypertensive individuals, with a significant association (p < 0.001). Moreover, 58.7% of hypertensive people and 39.8% of normotensive people mentioned having vision problems. By scientific standards, this difference was significant (p < 0.05). In summary, while several symptoms were common among both normotensive and hypertensive individuals, sleep apnea, abnormal heartbeat, and vision problems showed significant associations with high blood pressure.

## Discussion

HTN is a prevalent condition with a variety of hidden accompanying symptoms. In our research, we found that patients visiting community health centers have a significantly higher percentage of hypertensives compared to those with normotensives, among all hospital visitors seeking treatment for various medical concerns. This trend raises concerns, particularly within the context of healthcare in Pakistan. Hence, in order to manage or reduce the occurrence of high blood pressure, it is imperative to promptly enforce policies advocating for lifestyle adjustments and widespread educational initiatives.

The gender distribution was almost similar among hypertensives, with a slightly higher proportion of males. This finding aligns with studies in Pakistan [7] and the United States [8], whereas it is in contrast with findings from Bangladesh [9], showing diverse gender distributions among hypertensive individuals. Until puberty, blood pressure is similar in both genders, and afterward, it is significantly higher in males compared to age-matched females [10]. However, a higher prevalence of HTN is seen in women after 60 years of age [11]. Hypertensive individuals in the present study were, on average, older than normotensive ones, indicating age as a significant risk factor for HTN. This result is consistent with previous studies [7,12], highlighting age-related increases in HTN prevalence. We did not find any difference in BMI or respiratory rate between normotensives and hypertensives. The hypertensive group had a much slower heart rate (p < 0.05) than the normotensive group. Furthermore, we observed a significantly higher number of smokers present in hypertensive groups, indicating smoking is a risk factor for high blood pressure.

We investigated various symptoms in normotensive and hypertensive individuals. In our study, hypertensive individuals reported a higher percentage (79.9%) of headaches compared to normotensive individuals (71.6%). We find studies from Asian countries; for instance, India [13] and Bangladesh [14] have reported that headaches are common in hypertensive individuals. These findings suggest that headaches may be a common symptom associated with high blood pressure in the Asian population.

In our findings, we observed a higher percentage of vertigo, dyspnea, and chest pain in hypertensive individuals than in normotensives. A recent study indicated that dizziness and vertigo are prevalent among individuals with HTN, likely linked to vestibular issues [15]. It suggested clinicians should consider the possibility of HTN when patients complain of symptoms like vertigo, headaches, blurred vision, or chest pain during their initial visit. In our study, we found out that both normotensives and hypertensives reported a high percentage of fatigue, which is also indicated by other studies where HTN is linked with mild symptoms of dizziness, dyspnea, chest pain, headache, and fatigue [13,16].

In the present study, epistaxis (nosebleeds) was found to be uncommon in both groups. However, other studies suggest people with consistently high blood pressure are more likely to have repeated nosebleeds compared to those whose blood pressure only rises during the nosebleed itself [17,18]. We did not find a strong link between confusion and high blood pressure in this study; however, cognitive dysfunction in the elderly is associated with HTN [19], as we already demonstrated that age is also a significant risk factor for HTN.

A strong link was found between sleep apnea and high blood pressure. People who have sleep apnea are more likely to have high blood pressure. This might be because of inconsistent hypoxia, which causes more sympathetic activity and less baroreflex gain. Furthermore, another contributor is the stimulation of the renin-angiotensin-aldosterone system [20].

In the present study, the hypertensive group was shown to have a high number of patients with palpitations, which are abnormal heartbeats or skipped beats, as well as a sensation of unpleasant rapid pulsations in the chest area.

One study depicted that with increased SBP, there were diminished heart palpitations, and at SBP >180 mmHg, palpitations were abolished. Furthermore, hypertensive males exhibited increased chest pain and heart palpitations, whereas women reported headaches and nausea/vomiting [21].

The study found that people with high blood pressure had a considerably higher prevalence of vision issues than people with normal blood pressure (p < 0.05). One of high blood pressure’s most prevalent long-term indicators is visual impairment. It has an impact on someone’s independence, mobility, and quality of life. A recent study demonstrates 32.4% visual impairment among hypertensive patients with a duration of HTN ≥5 years [22]. These results emphasize the importance of regular eye examinations for hypertensive patients.

Limitations

The study has some limitations. First, as the data were collected at one point in time in the OPD, there was no follow-up on the cases studied. Second, because the study involved only two centers, the data may not be reflective of the whole population. Third, given the high prevalence of HTN, the sample size was relatively small.

## Conclusions

The prevalence of HTN is considerable in this part of the world. We found a high frequency of common symptoms in hypertensive individuals, such as vision problems, apnea, and palpitations. In summary, our study aligns with existing evidence from other Asian countries, emphasizing the relevance of these symptoms in hypertensive individuals. Strategies are required to diagnose and treat HTN at an early stage to avoid long-term, serious complications.
